# Prolonged Hypoxia in Rat Living Myocardial Slices Affects Function, Expression, and Structure

**DOI:** 10.3390/ijms26010218

**Published:** 2024-12-30

**Authors:** Florian J. G. Waleczek, Giuseppe Cipriano, Jonas A. Haas, Ankita Garg, Angelika Pfanne, Annette Just, Susanne Neumüller, Jan Hegermann, Andreas Pich, Ante Radocaj, Ke Xiao, Natalie Weber, Thomas Thum

**Affiliations:** 1 Institute of Molecular and Translational Therapeutic Strategies (IMTTS), Hannover Medical School, 30625 Hannover, Germany; waleczek.florian@mh-hannover.de (F.J.G.W.); cipriano.giuseppe@mh-hannover.de (G.C.); jonas.a.haas@stud.mh-hannover.de (J.A.H.); ankita.garg@bayer.com (A.G.); pfanne.angelika@mh-hannover.de (A.P.); just.annette@mh-hannover.de (A.J.); neumueller.susanne@mh-hannover.de (S.N.); xiao.ke@mh-hannover.de (K.X.); 2 Department of Cardiology and Angiology, Hannover Medical School, 30625 Hannover, Germany; 3 Institute of Functional and Applied Anatomy, Hannover Medical School, 30625 Hannover, Germany; hegermann.jan@mh-hannover.de; 4 Institute of Toxicology and Core Unit Proteomics, Hannover Medical School, 30625 Hannover, Germany; pich.andreas@mh-hannover.de; 5 Institute of Molecular and Cell Physiology, Hannover Medical School, 30625 Hannover, Germany; radocaj.ante@mh-hannover.de

**Keywords:** rat living myocardial slices, hypoxia, force generation, tissue model, gene expression, proteome

## Abstract

Ischemic heart disease is the leading cause of death worldwide. Reduced oxygen supply and myocardial hypoxia lead to tissue damage and impairment of the heart function. To the best of our knowledge, the primary functional effects of hypoxia in the multicellular model of living myocardial slices (LMSs) have not been investigated so far. In this study, we analyzed force generation, ultrastructure, gene expression, and proteome changes in rat LMS after 24 h of ex vivo culture in normal and reduced levels of oxygen (O_2_). We observed a significant reduction in absolute force and a slowdown of force kinetics as well as an increase in cardiomyocyte apoptosis and myofibrillar and mitochondrial damage, as well as transcriptomic changes. Proteome analysis revealed the deregulation of proteins involved in metabolic processes, hypoxic response, and neutralizing of reactive oxygen species. Our results indicate that hypoxia induces substantial primary changes in heart tissue, which are independent of perfusion and immune responses. Our new LMS model could serve as a screening system for drug development and new mechanistic insights.

## 1. Introduction

The heart is the organ that consumes the highest oxygen amount in the human body (~0.1 mL O_2_/g/min) and requires ~6 kg ATP per day [1]. Hypoxia arises when the oxygen demand is higher than its supply, and it can aggravate cardiovascular diseases such as ischemic heart disease, cardiac hypertrophy, hypertension, and heart failure [2]. Ischemic heart disease is the leading cause of death worldwide, affecting 126 million individuals (1655 per 100,000), approximately 1.72% of the world population [2]. This condition is characterized by chronic local hypoxia caused by various degrees of macro- and microvascular obstructions and impaired vascularization leading to decrease in oxygen supply [3], myocyte death, and activation of protective and detrimental downstream signaling pathways [4].

In hypoxic conditions, many genes and proteins are transcriptionally and translationally regulated [5]. One of the most investigated regulatory networks activated in hypoxic environments in the cardiac muscle is centered around hypoxia-inducible factor-1α (HIF-1α), which has been shown to have anti-apoptotic effects and directly targets the expressions of glycolytic enzymes influencing the energy supply of the cardiac muscle during hypoxia [6]. One major downstream target gene of HIF-1α is the vascular endothelial growth factor (VEGF), since it is crucial in the early response to hypoxia and the promotion of angiogenesis. Several microRNAs have been identified as responsive to hypoxia [7]. Among them, hypoxia-inducible microRNA 210 has been shown to improve angiogenesis and to inhibit apoptosis [8].

Investigating protective and damaging mechanisms during prolonged myocardial hypoxia and formulating possible therapeutic strategies involves in vitro and in vivo experiments. In vivo experiments require direct exposure of the whole animal to the reduced ambient O_2_ concentration or the occlusion of coronary arteries to cause myocardial ischemia, for example, in the LAD-ligation model [9]. Worsening of the heart function is then monitored at defined time intervals. Most importantly, hypoxia’s primary effects in the myocardium remain unresolved since fast migration and infiltration of the immune cells and the release of cytokines confound the detection [10,11]. Studying the primary reactions of the myocardial tissue to hypoxia is useful to gain mechanistic insights and to develop targeted cardioprotective therapies.

In vitro experiments do not require the induction of injury in animals but often do not recapitulate the whole heart’s complex multicellular composition and functional aspects [12]. Simple in vitro experiments provide results from cellular reactions to a hypoxic environment induced in the incubators or by using compounds depleting oxygen or stabilizing intracellular hypoxia-related signaling factors [13]. However, such cell cultures lack the capacity for multifaceted interactions and communication among cardiomyocytes and other cell types and usually lack mechanical stimulation (e.g., mechanical preload). Several complex models of myocardial hypoxia in cardiac organoids demonstrated their usability in this context [14]. Challenges arise when measuring the function of randomly organized cardiomyocytes in the organoids under undefined mechanical load conditions. In addition, in one of the most recent multilineage organoids, where both cardiac and gut cell lineages are co-cultured [15], cells organize in a spheroid with epicardial-like cells in the outer layer and cTnT^+^-cardiomyocytes in the core, surrounded by smooth muscle-like cells. This is reminiscent of a multi-chamber organization of a primitive heart, as indicated by a high presence of glycogen vesicles, the primary energy source in fetal hearts [16], and the expression of FGF10, which enhances cardiac specification and cardiomyocyte proliferation during heart development [17,18]. These organoids lack an adult mature phenotype, which could affect their response to hypoxia. Dissected primary papillary muscles provide another multicellular platform to study the cardiac muscle response to hypoxia [19]. However, due to the thickness of the tissue preparation, the experiments can be performed only on a short time scale.

Living myocardial slices (LMSs) bridge the gap between in vivo and in vitro cardiac models, being multicellular preparations with functional readouts [12]. LMS are precision-cut thin (100–400 µm) sections of the myocardium [20] and have been used previously to study reactions of resident cardiac macrophages to the increase in mechanical load in the LMS and responses of human LMS to novel drugs and non-coding RNA therapies [21,22,23]. The development of devices enabling long-term culture of LMS [24] with continuous recording of the contractile function allows researchers to investigate the mechanisms of cardiomyocyte function in a physiological or diseased state in the native environment over a time period from days to months.

So far, the functional reactions to hypoxia have not been studied in LMS during prolonged ex vivo culture. LMSs prepared from rat hearts can serve as a model to investigate pathological states. In this study, we asked to what extent primary responses to prolonged hypoxia affect the myocardial tissue in LMS regarding function, expression, and structure.

## 2. Results

### 2.1. Permanent Hypoxia Reduces Force Generation and Prolongs Relaxation in Rat Myocardial Slices

**Figure 1:**
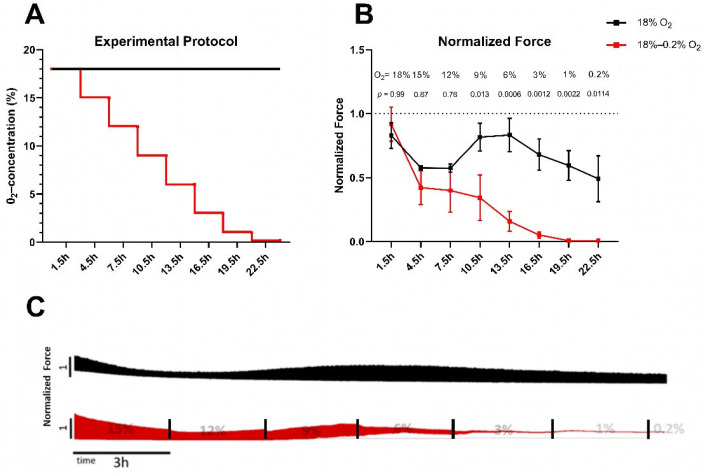
(**A**) Experimental protocol. *X*-axis: time; *y*-axis: O_2_ concentration (%). The control LMS (18% O_2_, black) were kept at 18% for the whole experiment. O_2_ concentration of the hypoxic LMS (9% O_2_, red) was lowered every three hours by 3% until it reached 3%, and then it was lowered to 1% and 0.2%. The stepwise decrease in O_2_ took from 30 sec (18–15%) up to 15 min (3–0.2%). During nitrogen insufflation, the lowest achieved CO_2_ concentration was 4.2%. (**B**) Normalized force was measured 1.5 h after the respective decrease in O_2_ concentration. *X*-axis: time; *y*-axis: force normalized to the force at 15 min (dotted line). Data are shown as mean ± SEM. Significance was tested with two-way pairwise ANOVA with Sidak post hoc test. N = 3 rats, n = 5 and 7 LMS for normoxic (black) and hypoxic (red) conditions, respectively. *p*-values are stated above data points. (**C**) Representative recordings of force of control LMS (top, black) and hypoxic LMS (bottom, red). *X*-axis: time; *y*-axis: force normalized to the force at 15 min.

To investigate the relation between force generation and oxygen concentration, we reduced O_2_ concentration in a stepwise manner and measured the force generation and force kinetics of rat LMS upon electrical stimulation. After the LMS were placed into the cultivation chambers, the experimental protocol was applied as indicated in Figure 1A. O_2_ concentration was rapidly decreased by 3% between the steps and kept for 3 h at the defined concentration, when force was measured. Figure 1B,C show the normalized force of the LMS at the decreasing hypoxic O_2_- concentrations and at a continuous normoxic O_2_- concentration.

With the reduction in O_2_ concentration, the normalized force of the rat LMS decreased. A significant difference of force between hypoxic and normoxic LMS was observed at 9% O_2_. Further reductions in O_2_ concentration (< 6%) resulted in a rapid decline in force generation. At 1% O_2_ concentration, the force decreased to unmeasurable values. Note a time-dependent reduction in force also in the normoxic LMS. Here, the force amplitude reached a local minimum after 7.5 h of ex vivo cultivation and recovered to 90% of the original value after 10.5 h. This phenomenon in ex vivo cultured rat LMS has been previously reported, and the reasons remain unexplored [22].

Because a significant reduction in normalized force was observed already at 9% O_2_ concentration, we investigated the effect of constant hypoxia of rat LMS at 9% O_2_ concentration for 24 h as has been previously applied in human cardiac organoids for the modeling of myocardial infarction [14].

**Figure 2:**
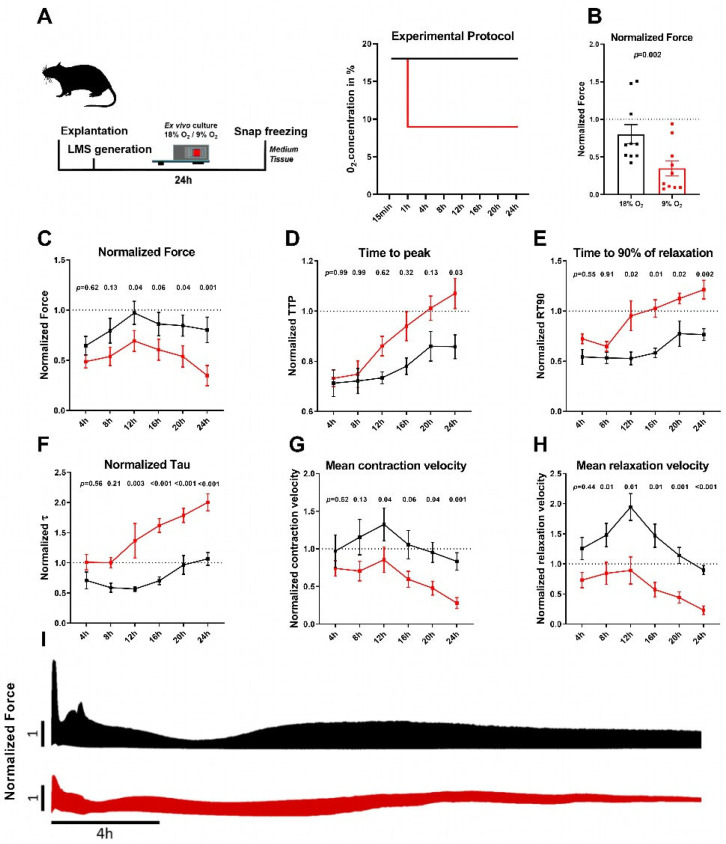
(**A**) Scheme showing experimental protocol. Diagram with *x*-axis: time; *y*-axis: O_2_ concentration (%) in normoxic (18% O_2_, black) or hypoxic (9% O_2_, red) LMS. O_2_ concentration of hypoxic LMSs was reduced after 1 h from 18% O_2_ to 9% O_2_. The decrease in O_2_ from 18% to 9% lasted 2 to 3.5 min. During nitrogen insufflation, the lowest achieved CO_2_ concentration was 4.2%. (**B**) Normalized force at 24 h (18% O_2_, black vs. 9% O_2_, red). *Y*-axis: relative force generation normalized to the force at 15 min (dotted line). Data are shown as mean ± SEM. Significance was tested with paired Student’s *t*-test; each dot represents the mean of the LMS generated from each rat; (N = 10 rats, n = 15 and 19 for normoxic and hypoxic LMS, respectively). (**C**–**H**) Contraction parameters of LMSs analyzed at indicated time points. *X*-axis: time, *y*-axis: parameter normalized to the value at 15 min (dotted line). Data are shown as mean ± SEM. Significance was tested with 2-way pairwise ANOVA with Sidak post hoc test. N = 9–10 rats, n = 15 and 19 for normoxic (black) and hypoxic (red) LMS, respectively. For absolute values refer to Supplementary Table S1. *p*-values are stated above the data points. (**C**) Normalized force, (**D**) time to peak (TTP), (**E**) time to 90% of relaxation (RT90), (**F**) decay constant of the relaxation (τ), (**G**) mean contraction velocity, and (**H**) mean relaxation velocity. (**I**) Representative force recordings of normoxic LMS (black, top) and hypoxic LMS (red, bottom). *X*-axis: time, *y*-axis: force normalized to force at 15 min (dotted line).

The LMS were subjected to 9% O_2_, and normalized force and kinetics were analyzed (Figure 2, Supplementary Table S1). We observed a significant reduction in the normalized force for hypoxic LMS compared with normoxic LMS after 24 h, while the first significant reduction occurred 12 h after the initiation of hypoxia (Figure 2B, C). In addition, time to peak (TTP, Figure 2D), time to 90% of relaxation (RT90, Figure 2E), and the decay time constant of relaxation (τ, Figure 2F), as well as the mean contraction and relaxation velocities (Figure 2G,H), were significantly altered for the hypoxic LMS at certain time points, indicating contractile systolic and diastolic impairments. Representative recordings of force generation over 24 h are shown in Figure 2I. We could not observe any relation between the functional parameters of the normoxic and hypoxic LMS and the age of the rats (9–12 weeks, Figure S1).

To investigate injury markers, we analyzed supernatants of cultured normoxic and hypoxic LMS. In the supernatant of hypoxic LMS, we measured higher concentrations of extracellular potassium, creatine kinase muscle–brain type (CK-MB), lactate dehydrogenase (LDH), and lactate concentrations and a lower glucose concentration than in the supernatant of normoxic LMS, indicating higher glucose consumption and the development of intracellular acidosis and increase in cell injury in the hypoxic LMS (Figure S2A–F).

### 2.2. Hypoxia Induces Myofibrillar Disruption and Mitochondrial Changes in Rat LMS

**Figure 3:**
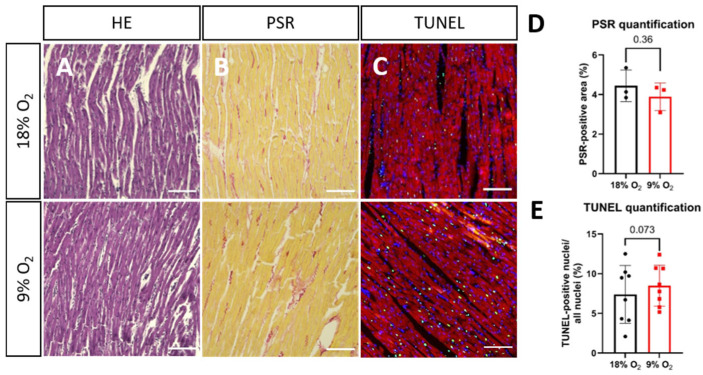
(**A**) Hematoxylin and eosin (HE) staining, (**B**) Picrosirius Red (PSR) staining, (**C**) TUNEL staining of rat LMSs. Vital nuclei are stained in blue, and apoptotic nuclei are stained in green; actin filaments are stained with phalloidin in red. Top row: normoxic LMSs, bottom row: hypoxic LMS. Scale bar 100 µm. (**D**) PSR-positive area as percentage of total area (mean ± SD, significance was tested with paired Student’s *t*-test, N = 3), (**E**) TUNEL-positive nuclei as percentage of all nuclei (mean ± SD, significance was tested with paired Student’s *t*-test, N = 8). Each dot represents the mean of two LMS generated from each rat (N = 3) in (**D**) and one LMS generated from each rat (N = 9) in (**E**).

For histological analysis, rat LMS were fixated after 24 h of ex vivo culture with 4% PFA, and histological sections of 5 µm were cut and stained with hematoxylin–eosin (HE) and Picrosirius Red (PSR). Representative images are shown in Figure 3. We could not detect any obvious alterations in tissue morphology in HE- and PSR-positive areas in hypoxic vs. normoxic LMS, indicating no changes in collagen deposition or degradation (Figure 3A,B,D). To evaluate if cellular death increased in hypoxic LMS, we performed apoptosis staining in histological sections from the LMS. Indeed, in hypoxic LMS, a higher fraction of cardiomyocytes was stained positive for apoptosis (Figure 3C,E).

**Figure 4:**
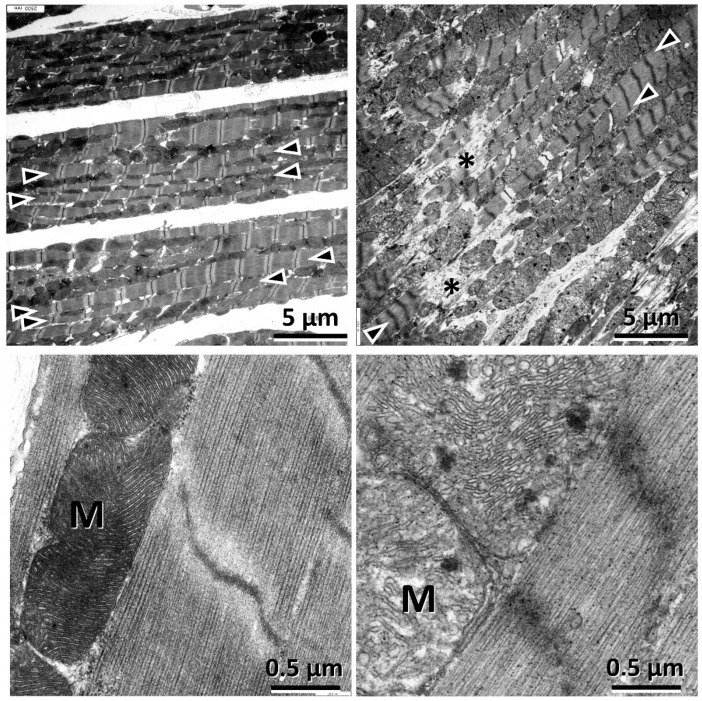
Transmission electron microscopy of LMS. Left: normoxic LMS; right: hypoxic LMS. LMS were cultured for 24 h and then fixated under pre-stretch to 2.1 µm sarcomere length. After embedding, sectioning was performed longitudinally to the cardiomyocyte alignment of the tissue, and the central region was analyzed. Note the aligned and uninterrupted myofibrils in the normoxic sample (**top left**, arrowheads), compared with the disconnected ones in the hypoxic sample (**top right**, arrowheads, disruptions marked by asterisks). Mitochondria profiles (M) in the normoxic sample appear more electron-dense than the rest of the tissue, with clearly visible and parallel continuous cristae (**bottom left**). In contrast, in the hypoxic sample, mitochondria appear less electron-dense and mostly swollen, with irregular cristae (**bottom right**).

To investigate the ultrastructure of the myofibrils and cellular organelles, we performed transmission electron microscopy (TEM) in hypoxic and normoxic LMS. After 24 h of ex vivo culture, we observed damage to the slice surface, presumably caused by the slicing procedure. However, the cell layers directly below the cutting surface showed well-organized and fully intact myofibrils with interspersed mitochondria (Figure 4, left side, myofibrils are indicated with the arrows; Figures S3 and S4). In contrast to this, the myofibrils of the hypoxic LMS were severely disrupted (Figure 4 right side and Figure S4). Mitochondria were damaged by a (partial) depletion of oxygen. While in normoxic LMS, we observed clearly visible and parallel, continuous mitochondrial cristae, in hypoxic LMS, mitochondria appeared less electron-dense, containing aggregates, and were mostly swollen with irregular cristae (Figure 4 bottom left vs. right; Figure S4). We conclude that prolonged hypoxia induced myofibrillar and mitochondrial damage in the hypoxic rat LMS.

### 2.3. Expressional Alterations in Hypoxic Rat Myocardial Slices

**Figure 5:**
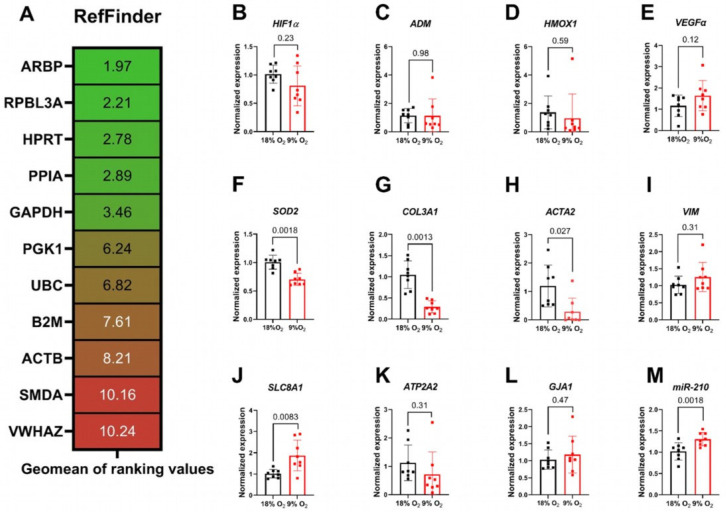
(**A**) Ranking of stability for housekeeper gene expressions determined by RefFinder, with *ARBP* being the most stable gene. (**B**–**M**) Normalized gene expressions in normoxic (black) and hypoxic (red) LMS. Gene expressions were normalized to the reference gene *ARBP* and the normoxic LMS (2^−ΔΔCT^ method). Hypoxia markers (*HIF1a*, *ADM*, *HMOX*, *VEGFa*, *SOD2*; (**B**–**F**)), fibrosis-associated/stromal cell markers (*COL3A1*, *a-SMA*, *VIM*; (**G**–**I**)), genes encoding for sodium/calcium exchanger NCX1 (*SLC8A1*) and SERCA2A (*ATP2A2*) and gap junction protein alpha 1 (*GJA1*; (**J**–**L**)) and hypoxia-related miR-210 (normalized to snRNA U6; M) were quantified between the groups. Data are shown as mean ± SD. Significance was tested with paired Student’s *t*-test. N = 4 rats, n = 8 and 8 for normoxic and hypoxic LMS, respectively; each dot represents one LMS. *p*-values stated above data points.

To investigate expressional changes in prolonged hypoxic conditions, we compared the expression levels of known reference genes after 24 h ex vivo culture in normoxic and hypoxic rat LMS: *ARBP*, *RPBL3A*, *HPRT*, *PPIA*, *GAPDH*, *PGK1*, *UBC*, *B2M*, *ACTB*, *SMDA*, and *VWHAZ* using RefFinder [25] (Figure 5A). *ARBP*, *RPBL3A*, and *HPRT* were the reference genes with the most stable expressions in hypoxic compared with normoxic LMS.

We further analyzed the mRNA expressions of Hypoxia-inducible factor 1-alpha (*HIF-1α*) and its downstream mediators Adrenomedullin (*ADM*) and Heme Oxygenase 1 (*HMOX1*) (Figure 5B–D). The expressions of these markers did not differ significantly between the hypoxic and normoxic LMS. *VEGFa*, a known hypoxia-sensitive gene [26], showed a slightly higher mean expression level in hypoxic rat LMS but without statistical significance (Figure 5E). In a hypoxic environment due to mitochondrial damage, reactive oxygen species (ROS) can be generated, leading to potential damage of the heart tissue [27]. We therefore analyzed the expression of Superoxide Dismutase 2 (*SOD2*, Figure 5F) as the ability of rat hypoxic slices to cope with the ROS production. Interestingly, the mRNA expression of this enzyme was downregulated in hypoxic rat LMS compared with normoxic LMS, indicating a reduction in anti-oxidative capacitance. We analyzed the expression of the fibrosis-associated genes Collagen Type III Alpha 1 Chain A1 (*COL3A1*) and smooth muscle actin (*ACTA2*) and the stromal cell marker Vimentin (*VIM*) (Figure 5G–I). The expressions of *COL3A1* and *ACTA2* were significantly reduced in hypoxic LMS. Downregulation of pro-fibrotic gene expressions could indicate a reduction in cardiac fibroblast numbers and a possible suppression of cardiac fibroblast activation and extracellular matrix production in a prolonged hypoxic environment in rat myocardial LMS. We measured functional impairment in force generation and in relaxation kinetics in the hypoxic rat LMS, which prompted us to further investigate the expression levels of sodium/calcium exchanger 1 (*SLC8A1*), sarcoplasmic/endoplasmic reticulum calcium ATPase 2 (*ATP2A2*), and electrical propagation gap junction protein alpha 1 (*GJA1*). We found a significant upregulation of *SLC8A1* expression; the other gene expressions were not significantly changed (Figure 5J–L). Upregulation of *SLC8A1* could be interpreted as a compensatory mechanism for cardiomyocytes to balance the intracellular calcium concentration during the hypoxic injury.

MicroRNA-210 is involved in cardiac ischemia [7,8,28,29] and can serve as a biomarker of cardiac injury in the ex vivo LMS model. Indeed, analysis of microRNA 210-3p expression revealed an upregulation of this microRNA in the hypoxic LMS (Figure 5M), suggesting its possible usage as a biomarker of hypoxic cardiac injury ex vivo.

### 2.4. Proteome Changes in Hypoxic LMS Reveal Alterations in Metabolic Pathways, Oxidative Stress, and Activation of Heat Shock Proteins

**Figure 6:**
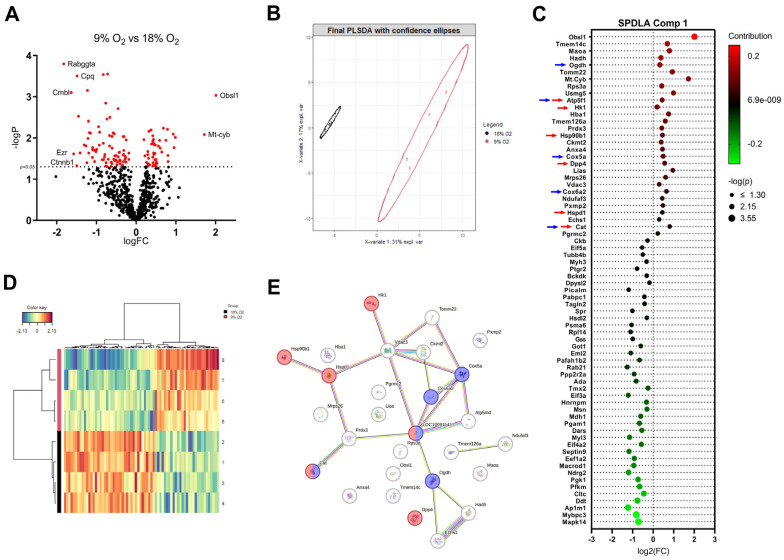
(**A**) Volcano plot of proteins in hypoxic LMS compared with normoxic LMS quantified with mass spectrometry (in total, 126 deregulated proteins). N = 4 rats, n = 4 and 4 normoxic and hypoxic LMS, respectively. (**B**) Component plot of PLSDA differentiating both conditions with confidence ellipses. (**C**) Bubble plot presenting the proteins contained in component 1 of the PLSDA. *X*-axis: log2(FC). Bubble color: contribution to the PLSDA component 1, bubble size: −log(p). Colored arrows: proteins in STRING clusters in (**E**). (**D**) Heatmap of the PLSDA model using 78 proteins. *X*-axis: proteins. *Y*-axis: normoxic (black) and hypoxic (red) LMS. (**E**) STRING network of upregulated proteins in component 1. Proteins enriched in the GO cluster “response to hypoxia” marked in red and those enriched in “aerobic respiration” marked in blue.

We investigated proteome changes in the hypoxic LMS compared with normoxic LMS. In total, we identified 969 proteins in the LMS, with 126 proteins significantly changed (*p* ≤ 0.05) (Figure 6A). The top 10 significantly upregulated proteins in hypoxic LMS based on fold-change were transmembrane protein 14C (Tmem14c), hemoglobin subunit α1 (Hba1), monoamine oxidase A (Maoa), catalase (Cat), cytochrome c oxidase subunit 7A2 (Cox7a2), translocase of outer mitochondrial membrane 22 (Tomm22), lipoic acid synthetase (Lias), upregulated during skeletal muscle growth protein 5 (Usmg5), mitochondrially encoded cytochrome B (Mt-Cyb), and obscurin like cytoskeletal adaptor 1 (Obsl1). The top 10 significantly downregulated proteins in the hypoxic LMS were rab geranylgeranyltransferase subunit alpha (Rabggta), carboxymethylenebutenolidase homolog (Cmbl), ezrin (Ezr), catenin beta 1 (Ctnnb1), carboxypeptidase Q (Cpq), isochorismatase domain containing 1 (Isoc1), glutathione s-transferase mu2 (Gstm2), acylaminoacyl-peptide hydrolase (Apeh), ras-related protein rab-21 (Rab21), and peptide methionine sulfoxide reductase (Msra) (Figure 6A).

To identify key pathways involved in the hypoxic response in our model, a PLSDA (Partial Least Squares Discriminant Analysis) was performed, resulting in a model using 78 proteins in two components with the goal of furthest distance of separation (Figure 6B,D). We further investigated the proteins of component 1, as they showed a significant separation on the *x*-axis. Of the 70 proteins in component 1 (Figure 6C), those which were increased in the 9% O_2_ group were analyzed with STRING analysis to further look for GO biological process cluster enriched terms. The protein network (Figure 6E) showed an enrichment in the terms: GO:0042744 (hydrogen peroxide catabolic process), GO:0009060 (aerobic respiration; red) GO:0006091 (generation of precursor metabolites and energy), and GO:0001666 (response to hypoxia, blue). STRING network analysis highlighted hexokinase 1 (Hk1), the first and rate-limiting enzyme of glycolysis, as upregulated in addition to several increased expressions of heat shock proteins and their binding partners (Hsp90b1, Hspd1), increased catalase (Cat), and other proteins responsible for energy production through aerobic respiration (Cox5a/Cox6q). We conclude that LMS undergo changes in metabolic, hypoxic, and ROS-neutralizing signaling pathways in prolonged hypoxic conditions.

## 3. Discussion

In this study, prolonged hypoxia was induced in adult rat LMS. We observed that cultivation of rat LMS in 9% O_2_ or less led to a significant reduction in force generation. Reduction in O_2_ concentration to 9% for 24 h reduced normalized force to 43% compared with normoxic LMS along with prolonged contraction and relaxation kinetics. In normoxic LMS, we found throughout the LMS thickness of 300 µm a well-structured myofibrillar network with well-preserved mitochondria, indicating sufficient oxygen diffusion throughout the LMS. However, impeded oxygen diffusion throughout the hypoxic LMS led to oxygen deficiency in the deeper cell layers and increased anaerobic glycolysis with excessive production of lactate and free hydrogen ions, causing intracellular acidosis. In our study, we detected increased extracellular lactate levels, fortifying this data interpretation. A decreased intracellular pH in cardiomyocytes is known to reduce the calcium sensitivity of the contractile apparatus and to decrease maximum force generation [30,31,32,33]; we therefore assume that the functional changes observed in our hypoxic LMS can be partially explained by this sequence of events. A decrease in LMS myofibrillar cross-section could also contribute to reduced force generation in our hypoxic LMS.

At the histological level, we observed an increase in the fraction of apoptotic cells in the hypoxic LMS. Transmission electron microscopy revealed disruption of myofibrils with a decrease in the myofibrillar cross-sectional area, which would contribute to the reduction in generated force in the hypoxic LMS. Moreover, mitochondria swelling was evident, possibly indicating severe mitochondrial damage, with increases in ROS generation and cytochrome b release from the damaged mitochondria into the cytosol. We speculate that mitochondrial damage could be accompanied by the activation of intracellular caspases and proteases with further proteolytic damage to myofibrils and cell apoptosis or necrosis [34]. This assumption is supported by our expressional data, which show dysregulation of catalase and LIAS and of superoxide dismutase in hypoxic LMS, indicating compensatory reactions, probably to the increased ROS production and mitochondrial damage in the hypoxic LMS.

In hypoxic conditions, many genes and proteins are transcriptionally and translationally regulated [5]. We screened a panel of potential reference genes [35,36,37,38] and compared their expression with RefFinder [25] for normoxic and hypoxic LMS. We found that *ARBP*, *RPBL3A*, and *HPRT* were stably expressed genes in both normoxic and hypoxic LMS. We next analyzed the relative expressions of the hypoxia-related genes *HIF1α* and *ADM* and found that they were not significantly altered in hypoxic compared with normoxic LMS. This could be due to the poor stability and fast degradation of *HIF1α* while briefly exposing the LMS to the normoxic environment upon tissue harvest. However, we could observe a slightly higher expression in hypoxic LMS of *VEGFa*, a downstream target of the HIF-1α factor. A higher expression of *VEGFa* could indicate an accumulation of HIF1α in the myocardium [26] and fortifies the suitability of our ex vivo hypoxia model. In acute severe hypoxia, it is known that cardiac fibroblasts undergo contractile activation and secrete larger amounts of extracellular material, e.g., collagens [39]. Therefore, we analyzed pro-fibrotic gene expressions in normoxic and hypoxic LMS. Surprisingly, we found downregulated levels of *COL3A1* and *ACTA2*, indicating that the cardiac fibroblast activation state is suppressed in our prolonged hypoxia model. Additionally, we speculate that cardiac fibroblasts in hypoxic LMS could also experience a hypoxic injury, reducing the fibroblast cell count.

Since we detected functional alterations in the hypoxic LMS, we hypothesize that functional genes involved in intracellular calcium homeostasis and electrical propagation also would be differentially regulated. We found an upregulation of the *SLC8A1* gene encoding the sodium–calcium exchanger, which could indicate a compensatory reaction to an increase in the intracellular calcium concentration in hypoxic LMS. It is known that intracellular sodium concentration can increase during acidosis, impeding the NCX1 exchanger function and leading to an increase in diastolic calcium level [40] and corresponding tissue damage. *SLC8A1* has been shown to be upregulated at the mRNA and protein levels in the hypoxic condition as a consequence of the suppressed activity of this transport system [41]. In line with this enhanced expression of NCX1 has been shown to reverse impaired functional phenotype in mouse hearts [42]. For the function-associated genes *ATP2A2* and *GJA1*, we did not find significant differences between the hypoxic and normoxic LMS.

Finally, we measured proteome changes in hypoxic LMS with enrichment in the categories for glycolysis, response to heat and hypoxia, and oxidative stress. We found differential protein expressions for heat shock proteins and their binding partners (Hsp90b1, Hspd1, Hsp90aa, Hspb1), presumably due to increased intracellular proteolysis with higher amounts of damaged and misfolded proteins in the hypoxic condition. Additionally, we found an increased protein level of obscurine-like 1 protein, a structural protein associated with titin and M-line [43]. This alteration could indicate sarcomere re-assembly and myofibrillar damage in hypoxia. Moreover, we measured microRNA-210-3p, a hypoxia-sensitive microRNA, and we found increased expression levels in hypoxic LMS, indicating its relevance as a potential biomarker in our prolonged hypoxia model.

We found hexokinase, a key player in the pathways for glycolysis, being upregulated. Based on the oxygen gradient across the LMS sections, we assume that cardiomyocytes in the core of the LMS mostly utilize anaerobic glycolysis, as indicated by the increased lactate production and increased glucose consumption in the supernatants of the hypoxic LMS. Increased LDH, extracellular potassium concentration, and CK-MB production in the supernatants of the hypoxic LMS also indicate increased tissue damage. However, we also observed an increase in the protein expression of some enzymes involved in oxidative phosphorylation processes in mitochondria [3], potentially indicating that different energy pathways become activated in our model in the hypoxic condition.

Overall, limitations of the LMS methodology include the removal of the myocardium from the circulatory system and absence of cellular and molecular interactions with the infiltrating immune system components, which could potentially protect or aggravate the hypoxic phenotype [44]. However, this model also allows the study of the primary effects of hypoxia in myocardial tissue. The response to a reduction in O_2_ concentration of different cell types in the heart can vary [45]. Observed effects in the multicellular LMS model cannot be entirely attributed to one cell type. Cell isolation from LMS with subsequent single-cell or single-nuclei sequencing approaches could provide detailed information about the responses of different cell types to the hypoxic environment.

Advantages of the ex vivo LMS hypoxia model include the possibility to perform multiple assays per slice and animal, reducing the number of sacrificed animals and avoiding in vivo experiments that would produce suffering and pain [46]. LMS preparations feature a multicellular environment [47], outperforming cellular in vitro models. The hypoxia LMS model could be adopted for large-mammal and human heart tissue slices for a more translational aspect in future studies. Using this model, deregulated genes and proteins can be identified, with the aim to open the possibility to design novel treatments for patients who have suffered damage in the myocardium due to hypoxic conditions. Moreover, different hypoxia induction and reoxygenation protocols could shed more light on the ischemia–reperfusion injury situation and disease development in patients with myocardial infarction [48].

## 4. Materials and Methods

### 4.1. Living Myocardial Slice Preparation and Force Measurement

The animal experiments in this study were authorized by the Institutional Animal Care and Research Advisory Committee and permitted by the responsible local authorities in Lower Saxony (LAVES, Oldenburg, Germany; 2022/293). The 9–12-week-old male Sprague-Dawley rats were housed in controlled humidity, temperature, and lighting conditions with food and water access ad libitum. LMS were generated from rat left ventricular tissue as described previously [22]. From each heart, 5–7 LMS could be prepared. The LMS were cultured ex vivo in biomimetic culture chambers (BMCCs, InVitroSys, Gräfelfing, Germany) under continuous electrical stimulation (maximum 3 V, 3–1–3 ms bipolar stimulation and 0.2 Hz to evaluate full relaxation property of the LMS), mechanical pre-stretch (12.6% of the resting slice length), and continuous agitation (60 rpm) [22,24]. Slices were cultured in 3 mL medium M199 (Sigma-Aldrich, St. Louis, MO, USA, #M4530) supplemented with ITS liquid media supplement (insulin, transferrin, selenite; Sigma-Aldrich, #I3146) in 1:1000 dilution and 3% penicillin/streptomycin (Thermo Fisher Scientific Inc., Waltham, MA, USA, #15140148). BMCC system permits continuous registration of LMS contractions, which were further processed with LabChart 8 Pro (ADInstruments Ltd., Oxford, UK). Force, calculated as maximum force minus baseline force; time to peak (TTP); time to 90% of relaxation (RT90); decay time constant of relaxation (τ); and mean contraction and relaxation velocities were calculated at intervals and normalized to the value at 15 min.

### 4.2. Ex Vivo Culture of Normoxic and Hypoxic LMS

LMS were selected randomly from each animal to avoid effects of transmural differences. LMS were cultivated in BMCC at 37 °C and 5% CO_2_. The hypoxia protocol started after all slices were placed into the bioreactors in the incubators (Binder GmbH, Tuttlingen, Germany). A concentration of 9% O_2_ was achieved through oxygen displacement by nitrogen. The stepwise decrease in O_2_ took from 30 s (18–15%) up to 15 min (3–0.2%). The decrease in O_2_ from 18% to 9% lasted 2 to 3.5 min. During nitrogen insufflation, the lowest achieved CO_2_ concentration was 4.2% before being adjusted to 5% again.

After 24 h, LMS tissue and medium supernatants were frozen in liquid nitrogen for gene expression and proteome analysis and supernatant analysis, or LMS tissue was fixated for histology/transmission electron microscopy, as described below. Then, 200 µL culture medium supernatant was analyzed for extracellular potassium (Cobas 8000, Modul ISE, Roche, Basel, Switzerland), creatine kinase muscle–brain type (CK-MB, Immunologic UV-Test, Cobas 8000, Modul c701, Roche), LDH (Cobas 8000, Modul c701, Roche), glucose (Cobas 8000, Modul c701, Roche), and lactate concentrations (Cobas 8000, Modul c502, Roche).

### 4.3. RNA Extraction and qRT-PCR

RNA was isolated from the frozen LMSs as described previously [22]. Extracted RNA was reverse transcribed to complementary (cDNA) in 13 µL of nuclease-free water. Then, 4 µL iScript Reaction Mix was mixed with 2 µL oligo-dT Primers and 1 µL iScript reverse transcriptase (Bio-Rad, Feldkirchen, Germany, #170-8897BUN), and cDNA was synthesized using a thermocycler (TProfessional Trio, Biometra Ltd., Jena, Germany) with the following settings: 90 min at 42 °C and 5 min at 85 °C, resulting in 20 µL of cDNA, which was diluted 1:5 with nuclease-free water. Finally, 2 µL cDNA was mixed with 5 µL SYBR Green reaction (Bio-Rad, #172-5006CUST), 0.025 µL iQ SYBR 20x Precision Blue RT-PCR Dye (Bio-Rad, #1725555), 0.05 µL ROX 1:50 (Thermo Fisher Scientific, #12223012), and 10 µM of specific high-purity salt-free primer pairs (HPSF, Eurofins, Luxembourg City, Luxembourg) diluted in 2.425 µL of nuclease-free water. The following settings were used for the qPCR reaction: 95 °C for 3 min of pre-dissociation, 45 cycles, 95 °C for 15 s, then 60 °C for 30 s, and finally, 72 °C for 40 s. For microRNA quantification, Taqman assays were used according to manufacturer instructions (hsa-miR-210-3p, Thermo Fisher Scientific, Assay ID: 000512; U6 snRNA, Thermo Fisher Scientific, Assay ID: 001973). Primer sequences are given in Table 1. We analyzed gene expression changes by the 2^−ΔΔCT^ method normalized to the reference gene expression as determined by RefFinder (*ARBP*) [25].

### 4.4. Histology

For histological analysis, LMSwere fixated with 4% PFA for 30 min at RT and stored in phosphate-buffered solution containing 0.2 M sodium dihydrogen phosphate monohydrate (Roth, Bavaria, Germany, #4357) and 0.2 M di-sodium-hydrogen phosphate dehydrate (Sigma-Aldrich, #13472-35-0) in a ratio of 1:3.16 adjusted to pH 7.2–7.3. Samples were paraffinized, sectioned, and rehydrated using xylol-isopropanol-H_2_O. Samples were washed with water and stained for 8 min with filtrated DELAFIELD’s Hematoxylin solution (Chroma Waldeck, Muenster, Germany, #2C-161). The samples were rinsed for 8 min with water and stained for 3 min in 0.1% Eosin solution (Eosin G 0.5% Roth; # X883; 1:5 dilution in H_2_O) supplemented with three drops of glacial acetic acid (Roth, #3738). We used Picrosirius Red (PSR) staining to determine collagen content. After tissue fixation, tissue was rinsed with water and stained for 30 min with PSR staining solution containing 0.1 g Siriusred (Chroma Waldeck, #1A-280) dissolved in 100 mL of picric acid 1.2% (Chroma Waldeck, #3E-086). After rinsing with water, the slices were dehydrated in ascending alcohol dilutions with 70–90–100% isopropanol (2-Propanol, Roth, #6752.4), and then with xylol (ROTIPURAN^®^ Roth, #4436.2), and mounted with Eukitt mounting medium (Sigma-Aldrich, #03989-100). The images were acquired with Keyence microscope (BZ-X800) with 4×, 10×, and 20× objectives (Nikon, Tokyo, Japan, Plan Fluor). Images were processed with Fiji (version 1.54f, RRID:SCR_002285) [49].

### 4.5. TUNEL Staining

For TUNEL staining, LMS were fixated and processed as for histology, until the rehydration step. After being washed 4 times in PBS, the samples were stained with Click-iT™ Plus TUNEL-Assay-Kit Alexa Fluor 488 (Thermo Fisher Scientific, C10617) following the instruction of the manufacturer. Briefly, samples were permeabilized with Proteinase K for 15 min, then incubated for 10 min at 37 °C in a humidified jar with TdT reaction buffer and later for 1 h with TdT reaction mixture. After being washed with PBS, samples were incubated with Click-iT Plus TUNEL reaction mixture for 30 min at 37 °C, and then stained with Phalloidin antibody (Abcam, Cambridge, UK, ab176757) diluted 1:1000 in PBS for 1 h. After the last incubation for 10 min with Hoechst 33342 (Thermo Fisher, #H3570) diluted 1:1000 in PBS, samples were washed 3 times with PBS, and the coverslips were mounted using ProLong™ Gold antifade reagent (Invitrogen, Carlsbad, CA, USA, P36930). Images were acquired with Keyence microscope and quantified using Fiji.

### 4.6. Transmission Electron Microscopy

For transmission electron microscopy (TEM), LMS were cultured ex vivo under normoxic (18% O_2_) or hypoxic (9% O_2_) conditions for 24 h. The LMS were pre-stretched to 2.1 µm sarcomere length in a metal frame and fixated with TEM-fixative (1.5% formaldehyde, 1.5% glutaraldehyde in 150 mM/L HEPES, pH 7.35) for one hour at room temperature and left overnight at 4 °C. On the following day, the metal frame was removed, and the LMS were processed and embedded as described previously [50]. Ultrathin sections (60 nm) were cut parallel to the myocyte alignment.

### 4.7. Protein Extraction and MS Analysis of Proteins

Whole LMS were homogenized in a mixture of cell lysis buffer (Cell Signaling Technology, Danvers, MA, USA, #9803) and pefabloc SC (Sigma-Aldrich, #76307) for 20 s at 5000 rpm using Precellys24 (VWR, Radnor, PA, USA). We pooled two technical replicates per each rat heart and condition (hypoxic or normoxic LMS) prepared from four rat hearts. Protein was mixed with Laemmli buffer and incubated for 5 min at 95 °C. Proteins were then alkylated by addition of acrylamide up to a concentration of 2% and incubated at RT for 30 min. SDS PAGE was performed on 12% gels in a mini protean cell (Biorad) [51].

After electrophoresis, proteins were stained with Coomassie Brilliant Blue (CBB) for 15 min and background staining was reduced with water. Each lane was cut into four pieces, which were further minced to 1 mm^3^ gel pieces. Further sample processing was performed as described [51]. Briefly, gel pieces were destained two times with 200 µL 50% ACN, 50 mM ammonium bicarbonate (ABC) at 37 °C for 30 min and then dehydrated with 100% ACN. Solvent was removed in a vacuum centrifuge, and 100 µL 10 ng/µL sequencing grade Trypsin (Promega, Madison, WI, USA) in 10% ACN, 40 mM ABC were added. Gels were rehydrated in trypsin solution for 1 h on ice and then covered with 10% ACN, 40 mM ABC. Digestion was performed overnight at 37 °C and was stopped by adding 100 µL of 50% ACN, 0.1% TFA. After incubation at 37 °C for 1 h, the solution was transferred into a fresh sample vial. This step was repeated twice, and extracts were combined and dried in a vacuum centrifuge. Dried peptide extracts were redissolved in 30 µL 2% ACN, 0.1% TFA with shaking at 800 rpm for 20 min. After centrifugation at 20,000× *g,* aliquots of 12.5 µL each were stored at −20°C.

### 4.8. LC-MS Analysis

Peptide samples were separated with a nano-flow ultra-high-pressure liquid chromatography system (RSLC, Thermo Scientific) equipped with a trapping column (3 µm C18 particle, 2 cm length, 75 µm ID, Acclaim PepMap, Thermo Scientific) and a 50 cm long separation column (2 µm C18 particle, 75 µm ID, Acclaim PepMap, Thermo Scientific). Peptide mixtures were injected, enriched, and desalted on the trapping column at a flow rate of 6 µL/minute with 0.1% TFA for 5 min. The trapping column was switched online with the separating column, and peptides were eluted with a multi-step binary gradient: linear gradient of buffer B (80% ACN, 0.1% formic acid) in buffer A (0.1% formic acid) from 4% to 25% in 30 min, 25% to 50% in 10 min, 50% to 90% in 5 min, and 10 min at 90% B. The column was reconditioned to 4% B in 15 min. The flow rate was 250 nL/minute, and the column temperature was set to 45 °C. The RSLC system was coupled online via a Nano Spray Soure II (Thermo Scientific) to Orbitrap Exploris 240 mass spectrometer. Metal-coated fused-silica emitters (SilicaTip, 10 µm i.d., New Objectives, Woburn, MA, USA) and a voltage of 2.1 kV were used for the electrospray. Overview scans were acquired at a resolution of 120k in a mass range of *m*/*z* 300–1500. Precursor ions of charges two or higher and a minimum intensity of 4000 counts were selected for HCD fragmentation with a normalized collision energy of 38.0, an activation time of 10 ms, and an activation Q of 0.250. Active exclusion was set to 70 s within a mass window of 10 ppm of the specific *m*/*z* value. Mass tolerances for precursor and fragment ions were 5 ppm and 0.3 Dalton, respectively.

Raw MS data were processed using Max Quant software (version 2.0, [52]), including search engine Andromeda and Perseus software (version 2.0.6.0, [53]) and rat entries of UniProt DB (93,042 entries, date: 07/2023). Proteins were stated identified with target-decoy base search by a false discovery rate of 0.01 on protein and peptide level. Allowed number of missed and/or non-specific cleavage was two. Variable modifications were deamidation on N, Q, oxidation on methionine; fixed modification: propionamidation on cysteine residues. For quantification of multiple isoforms, proteins were combined to protein groups according to Max Quant protein search.

### 4.9. PLSDA and STRING Enrichment Analysis of the Proteome Data

The protein quantification results were analyzed with R (version 3.6.0+) [54], RStudio (version 2024.09.1 + 394.pro7) [55], and the mixOmics [56] library to generate a model of best separation using a PLSDA. The data were scaled and a PLSDA model (“Mfold” validation, 50 repeats, measure “BER”, 2 components, 10–100 proteins per component, step size 2) was generated. Components plots with confidence ellipses and a heatmap of the final model were generated. The component details and contributing proteins were exported and visualized. The proteins enriched in the 9% O_2_ group were analyzed with STRING http://string-db.org (accessed on 21 September 2024) [57] protein analysis, interesting significantly enriched GO-clusters selected, and the bitmap exported (FDR < 0.05).

### 4.10. Statistical Analysis

Data were processed and are presented as mean ± SEM, if not otherwise stated. GraphPad Prism (version 9.1, RRID: SCR_002798, GraphPad Software Inc., La Jolla, CA, USA) was used for statistical tests and graph presentations. Data were tested for normal distribution. A statistical two-sided Student’s *t*-test, parametric ANOVA with post hoc Tukey’s for multiple comparisons, or two-way pairwise ANOVA with post hoc Sidak test was performed if normality was assumed. If normality was not assumed, Mann–Whitney or Kruskal–Wallis tests were used. The *p*-values ≤ 0.05 were considered statistically significant. Details on the applied statistical tests, number of animals (N, rats), and the number of normoxic or hypoxic LMSs (n) are given in the respective figure legends.

## 5. Conclusions

We studied the effects of prolonged hypoxia in rat living myocardial slices (LMS). We detected reductions in absolute force and slower relaxation kinetics for hypoxic vs. normoxic LMS after 24 h of ex vivo culture. Hypoxic LMS exhibited disruption of myofibrils and mitochondrial damage. In expression and proteome analysis, we found enriched pathways for metabolism, hypoxia, and ROS neutralization. Our study demonstrates that prolonged hypoxia induces direct primary effects in multicellular heart preparations, even though influences from the circulatory system and invading immune cells are excluded. LMS can serve as a multiparametric screening platform for drug candidates as well as for further investigations into myocardial hypoxia.

## Figures and Tables

**Table 1:** Primer sequences.

Gene	Forward 5′-3′ Primer Sequence	Reverse 5′-3′ Primer Sequence
*ACTB*	CTGAGGAGCACCCTGTGCTG	CCAGAGGCATACAGGGACAA
*ACTA2*	CATCACCAACTGGGACGACA	TCCGTTAGCAAGGTCGGATG
*ADM*	AATGAAGCTGGTTTCCATCG	TTAGCGCCCACTTATTCCAC
*ARBP*	AAAGGGTCCTGGCTTTGTCT	GCAAATGCAGATGGATCG
*ATP2A2*	AAGCCACAGAGACTGCTCTCAC	CGCAGAATCTTCCAGGTGCATC
*B2M*	TGTCTCAGTTCCACCCACCT	ATTACATGTCTCGGTCCCAGG
*COL3A1*	GAAACCCCAGCAAAACAAAA	TATTGGTGGGTGAAACAGCA
*GAPDH*	GAAGGGCTCATGACCACAGT	GGATGCAGGGATGATGTTCT
*GJA1*	CGCCGGCTTCACTTTCATTA	ATGAAGAGCACTGACAGCCA
*HIF1A*	GCCTCTGAAACTCCAAAGCC	ACGTTCCAATTCCTGCTGCT
*HMOX1*	CACGCATATACCCGCTACCT	AAGGCGGTCTTAGCCTCTTC
*HPRT*	CAGTCAACGGGGGACATAA	GCTGTACTGCTTGACCAAGG
*PGK1*	CCAGTCTAGAGCTCCTGGAAGGTAA	ATCCCGATGCAGTAAAGACGAG
*PPIA*	TATCTGCACTGCCAAGACTGAGTG	CTTCTTGCTGGTCTTGCCATTCC
*RPBL3A*	GCGGAGGGGCAGGTTCTA	CGAGACGGGTTGGTGTTCAT
*SDHA*	TACTGTTGCAGCACAGGGAG	CAGTCAGAGCCTTTCACGGT
*SLC8A1*	AGGTCCATGCTAGAGATCATCC	CCTCTCCTCCTCCTCTTTGC
*SOD2*	AAAGGAGAGTTGCTGGAGGC	CCTGAACCTTGGACTCCCAC
*UBC*	TCGTACCTTTCTCACCACAGTATCTAG	GAAAACTAAGACACCTCCCCATCA
*VEGFa*	ACGGGCCTCTGAAACCATGA	GCAGCCTGGGACCACTTG
*VIM*	GGGAGGAGAGCAGGATTTCT	TCATCGTGGTGCTGAGAAGT
*VWHAZ*	GATGAAGCCATTGCTGAACTTG	GTCTCCTTGGGTATCCGATGTC

## Data Availability

The original data are available here: https://doi.org/10.25625/WKWMNW.

## Supplementary Materials

The following supporting information can be downloaded at: https://www.mdpi.com/article/10.3390/ijms26010218/s1.
