# Supplementary material for: Prolonged Hypoxia in Rat Living Myocardial Slices Affects Function, Expression, and Structure
Source: Int J Mol Sci. 2024 Dec 30;26(1):218. doi: 10.3390/ijms26010218 (PMC11720517; doi:10.3390/ijms26010218)
Supplement: Supplementary file 1 [file ijms-26-00218-s001.zip › ijms-3382143-supplementary.pdf]

International Journal of *Molecular Sciences*

MDPI

Article

Prolonged hypoxia in rat living myocardial slices affects function, expression, and structure

Florian J. G. Waleczek [1,2,†], Giuseppe Cipriano [1,†], Jonas A. Haas [1], Ankita Garg [1], Angelika Pfanne [1], Annette Just [1], Susanne Neumüller [1], Jan Hegermann [3], Andreas Pich [4], Ante Radocaj [5], Ke Xiao [1], Natalie Weber [1,*,‡] and Thomas Thum [1,*,‡]

Supplemental Tables, Figures and Legends:

Suppl. Table 1:

	**Mean ± SD; (N/n)**	**Mean ± SD; (N/n)**
**Active force [mN]**	**18% O_2**	**9% O_2**
15min	10.76 ± 5.19; (10/15)	10.6 ± 3.8; (10/19)
4h	6.12 ± 3.06; (10/15)	4.77 ± 2.01; (9/16)
8h	6.9 ± 2.7; (10/15)	5.39 ± 1.42; (9/16)
12h	9.33 ± 4.89; (10/15)	6.02 ± 2; (9/16)
16h	8.84 ± 5.67; (10/15)	5.17 ± 2.29; (9/16)
20h	8.5 ± 5.71; (10/15)	5.07 ± 2.61; (9/16)
24h	7.84 ± 6.78; (10/15)	4.09 ± 3.58; (10/19)
**TTP [ms]**	**18% O_2**	**9% O_2**
15min	74.92 ± 20.9; (10/15)	69.99 ± 8.5; (10/19)
4h	52.68 ± 5.8; (10/15)	50.76 ± 7.72; (9/16)
8h	52.98 ± 7.62; (10/15)	51.69 ± 11.56; (9/16)
12h	57.26 ± 7.72; (10/15)	58.96 ± 4.7; (9/16)
16h	59.71 ± 9.63; (10/15)	64.36 ± 6.85; (9/16)
20h	62.55 ± 10.29; (10/15)	69.15 ± 9.19; (9/16)
24h	64.97 ± 9.39; (10/15)	73.81 ± 12.74; (10/19)
**RT90 [ms]**	**18% O_2**	**9% O_2**
15min	170.97 ± 38; (10/15)	144.94 ± 39.1; (10/19)
4h	84.6 ± 22.38; (10/15)	96.26 ± 22.19; (9/16)
8h	86.42 ± 23.57; (10/15)	91.37 ± 26.41; (9/16)
12h	94.61 ± 30.25; (10/15)	121.45 ± 30.19; (9/16)
16h	103.08 ± 30.88; (10/15)	137.61 ± 21.93; (9/16)
20h	115.06 ± 35.78; (10/15)	158.14 ± 42.43; (9/16)
24h	129.05 ± 43.52; (10/15)	171.13 ± 46.78; (10/19)
**τ [s]**	**18% O_2**	**9% O_2**
15min	0.063 ± 0.017; (10/15)	0.046 ± 0.008; (10/19)
4h	0.036 ± 0.013; (10/15)	0.05 ± 0.015; (9/16)
8h	0.035 ± 0.012; (10/15)	0.045 ± 0.012; (9/16)
12h	0.037 ± 0.017; (10/15)	0.061 ± 0.027; (9/16)
16h	0.043 ± 0.013; (10/15)	0.069 ± 0.018; (9/16)
20h	0.048 ± 0.017; (10/15)	0.079 ± 0.019; (9/16)
24h	0.057 ± 0.02; (10/15)	0.09 ± 0.022; (10/19)

Suppl. Table 1.

Force, contraction and relaxation parameters in normoxic and hypoxic LMS. Data is shown as mean±SD. Active force: force amplitude [mN]; TP100: time to peak in [ms]; RT90: time to 90% of relaxation in [ms]; τ: decay time constant of relaxation (starting from 50% of relaxation) in [s]; N=number of animals; n=number of LMS.

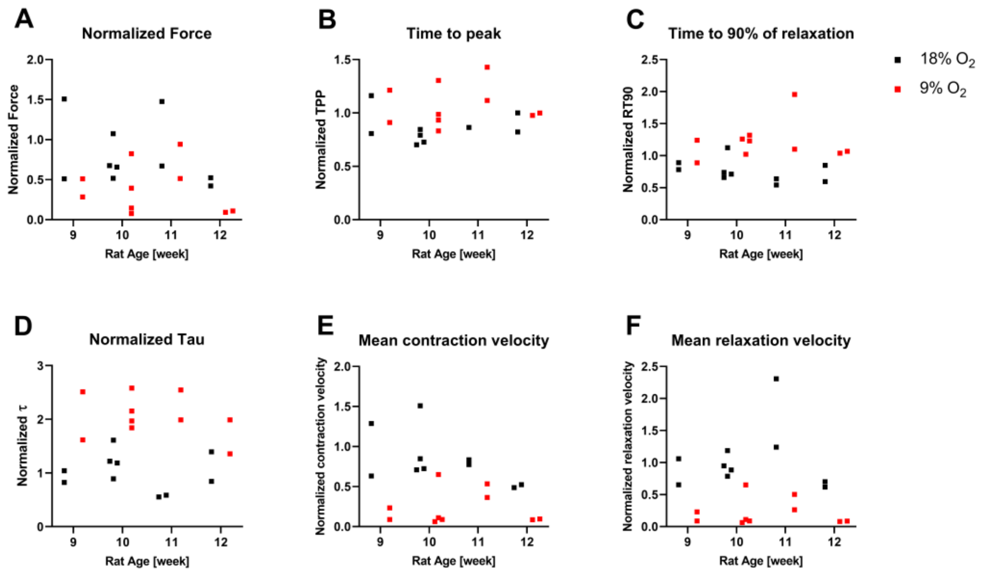

Figure S1.

A. Normalized force, **B.** Normalized time to peak, **C.** Normalized time to 90% of relaxation, **D.** Normalized Tau, **E.** Normalized mean contraction velocity, **F.** Normalized mean relaxation velocity in LMS prepared from rat hearts of different ages (9-12 weeks old) and cultivated in 18% O_2 (black) or 9% O_2 (red) for 24 h. Individual dots indicate mean values for each rat. No obvious age dependency of any of the six measured parameters is visible.

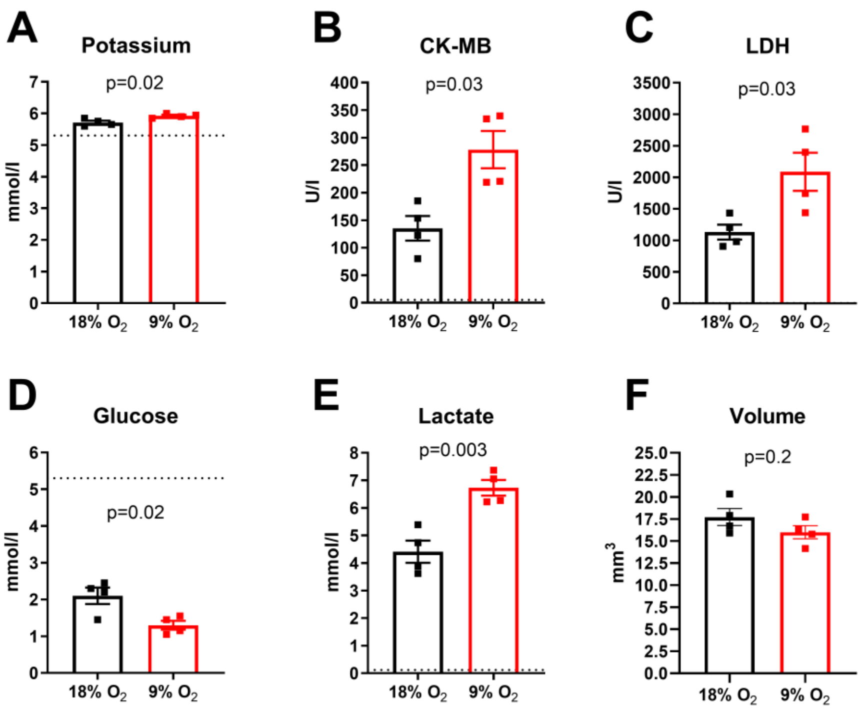

Figure S2.
Supernatants from normoxic (black) and hypoxic (red) LMS. **A.** Extracellular potassium (mmol/l), **B.** CK-MB (creatine kinase-MB, U/l), **C.** LDH (U/l), **D.** glucose (mmol/l) and **E.** lactate (mmol/l) concentrations were significantly elevated in the hypoxic LMS as compared to the normoxic LMS. **F.** Calculated volume (length×width×height) of the analyzed LMS. N=4, n=8 and 8 normoxic and hypoxic LMS, respectively. Data is presented as mean±SEM. Significance was tested with Student´s t-test. The dotted lines indicate concentrations measured in the fresh medium before LMS culturing. Numbers are p-values.

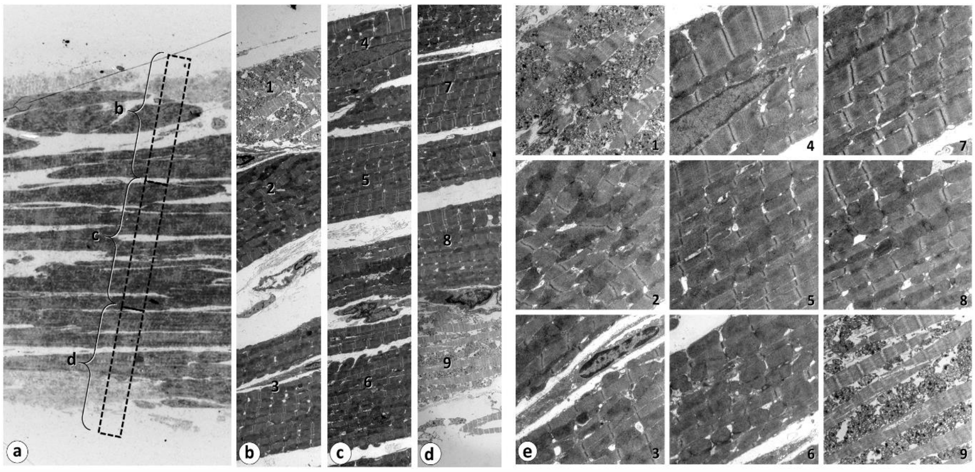

Figure S3.
Transmission electron microscopy of a complete section of normoxic LMS. **a**. Transverse section of complete LMS. **b, c, d.**, Higher magnification of the respective regions. **e.** Regions labelled with 1-9 at higher magnification. Note the cells at the top and at the bottom of the LMS are necrotic, while the cells in the core appear intact, with clearly visible myofibrils and mitochondria.

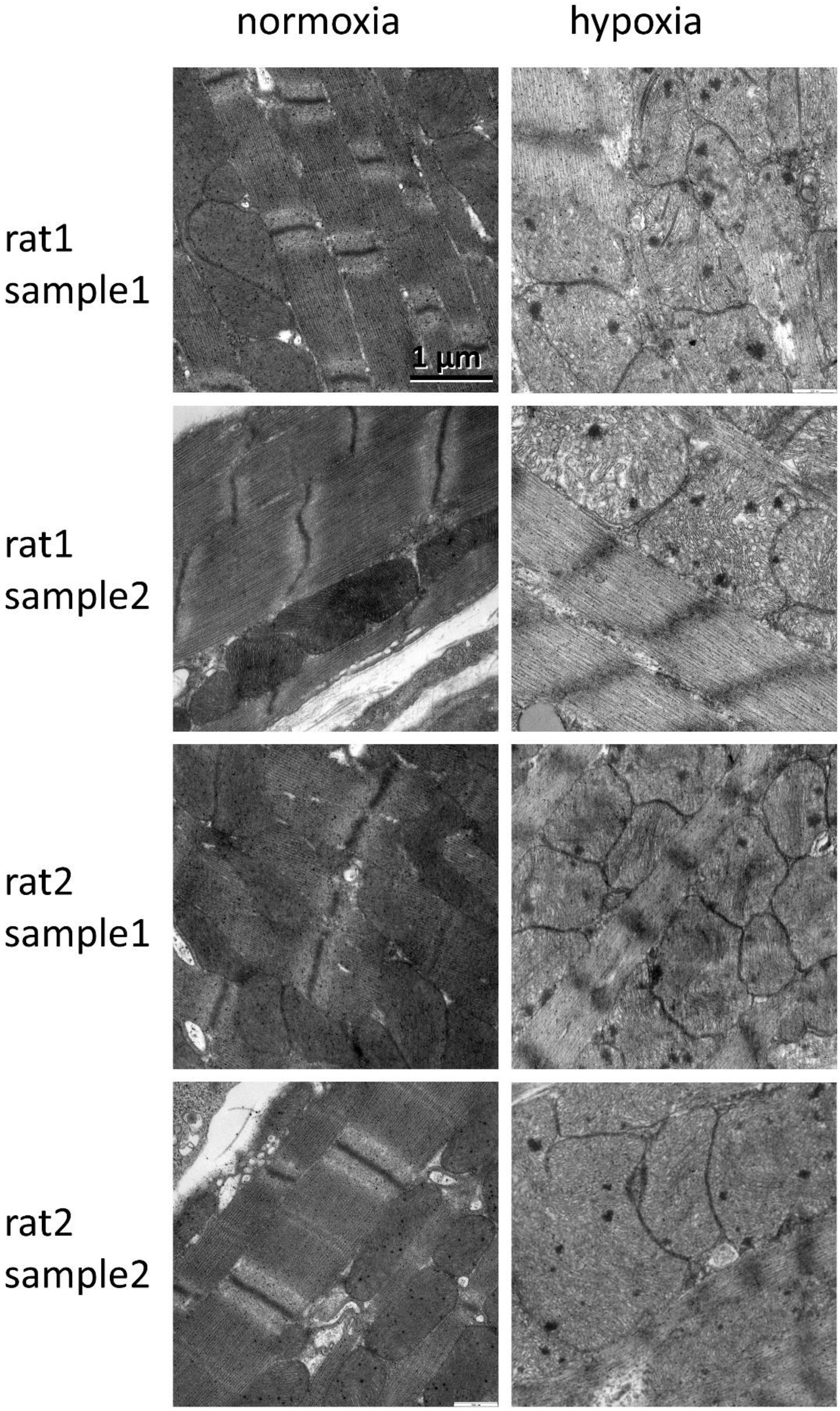

Figure S4.
Transmission electron microscopy of rat LMS (N=2) cultured for 24 hours *ex vivo*. Left: normoxic LMS; right: hypoxic LMS. Tissue samples from *ex vivo* culture were prestretched in the metal frame and fixated with the TEM fixans for one hour at room temperature and then overnight at 4°C. After embedding, sectioning was done longitudinally to the contraction-direction of the tissue and the core region was analyzed.
